# Hyperthyroidism induced by paraneoplastic human chorionic gonadotropin (hCG) production from testicular tumours: a retrospective clinical and histopathological study

**DOI:** 10.1530/EC-24-0341

**Published:** 2024-12-20

**Authors:** Julia Rohayem, Jan Idkowiak, Sebastian Huss, Thomas Balke, Hendrik Schürmann, Birthe Heitkötter, Joachim Wistuba, Angela Huebner

**Affiliations:** ^1^ Centre of Reproductive Medicine and Andrology, University Hospital Münster, Münster, Germany; ^2^ Children’s Hospital of Eastern Switzerland, St. Gallen, Switzerland; ^3^ Department of Metabolism and Systems Science, College of Medicine and Health, University of Birmingham, Birmingham, United Kingdom; ^4^ Centre for Endocrinology, Diabetes and Metabolism, Birmingham Health Partners, University of Birmingham, Birmingham, United Kingdom; ^5^ Department of Endocrinology and Diabetes, Birmingham Children’s Hospital, Birmingham Women’s and Children’s NHS Foundation Trust, Birmingham, United Kingdom; ^6^ Institute of Pathology, University Hospital Münster, Münster, Germany; ^7^ Department of Medical Oncology, University Hospital Essen, Essen, Germany; ^8^ Children’s Hospital, Universitätsklinikum Carl Gustav Carus Dresden, Technische Universität Dresden, Dresden, Germany

**Keywords:** hCG, hyperthyroidism, thyroid storm, thyrotoxicosis, testicular germ cell tumours, choriocarcinoma, embryonal carcinoma

## Abstract

Human chorionic gonadotropin (hCG) has structural similarities with thyroid-stimulating hormone (TSH) and may stimulate TSH receptors at higher concentrations. During pregnancy, placental hCG causes TSH suppression, contributing to hyperemesis. However, in males, clinical manifestations caused by excess hCG are rare. Herein, we describe complications of life-threatening thyroid storm caused by paraneoplastic hCG secretion from testicular germ cell tumours (GCTs) and aim to identify high-risk groups through retrospective analysis in *n* = 20 males (aged 17–55 years) with testicular hCG-positive GCTs. Seven hCG-positive testicular GCTs were classified as seminoma, and 13 were classified as non-seminomatous GCTs (NSGCTs). In 3/7 males with seminomas (43%), serum β-hCG concentrations were mildly elevated (median: 0.3 U/L; range: 0.3–82.1 U/L). In contrast, β-hCG was increased in 12/13 (92%) males with a NSGCT (median: 71.1 U/L; range: 0.3–1,600,000 U/L). In 10/13 males with NSGCT (77%), we detected components of embryonal cell carcinoma (EC), and in 7/13 (54%), we detected components of a choriocarcinoma (ChC). TSH was suppressed with high free thyroxine levels in two cases with NSGCT and excessively elevated β-hCG concentrations, but there was no TSH suppression in a further case with high β-hCG. One patient with NSGCT and high β-hCG levels presented with thyroid storm and imminent decompensation refractory to anti-thyroid treatment, requiring a total thyroidectomy. In the second patient, anti-thyroid treatment was initiated shortly after the diagnosis, successfully normalizing hyperthyroxinaemia. In conclusion, paraneoplastic β-hCG production, occurring in NSGCTs with components of ECs or ChCs, is a rare cause of thyrotoxicosis. Early recognition and treatment are critical to prevent a life-threatening thyroid storm.

## Introduction

Among adult carcinomas arising from the testes, malignant germ cell tumours (GCTs) comprise the vast majority (99%), with an incidence peak in the third to fourth decade of life. Germ cell tumours originate in primordial germ cells and are classified as seminomatous or non-seminomatous (NS) tumours. In 5% of males, GCTs derive from extragonadal tissues. NSGCTs may be composed of embryonal carcinomas (EC), teratomas (T), choriocarcinomas (ChC) or yolk sac tumours (YS) (1).

Elevated serum human chorionic gonadotropin (β-hCG) and alpha-fetoprotein (AFP) concentrations are present in about 60% of all males with NSGCTs, while β-hCG is only increased in serum in about 20% of men with seminomas. Lactate dehydrogenase (LDH) is a non-specific marker that correlates with the prognosis of metastatic stages (2).

ECs contain the most undifferentiated cell type in testicular tumours. ECs may produce highly elevated serum concentrations of β-hCG, AFP or both.

ChCs consist of cytotrophoblasts and syncytiotrophoblasts. Pure ChCs are rare and usually represent a widely metastatic disease, giving rise to high serum concentrations of β-hCG (1).

hCG belongs to a family of secreted glycoproteins, together with TSH, luteinizing hormone (LH) and follicle-stimulating hormone (FSH). All these peptide hormones are heterodimers, consisting of two non-covalently joined subunits: a common α- and a hormone-specific β-subunit. The β-subunit of hCG resembles the β-subunit of LH and, to a lesser degree, TSH. For both LH and hCG, signal transduction is facilitated by the LH/CG receptor. Both hCG and TSH β-subunits possess 12 highly conserved cysteine residues (3), forming a similar tertiary cystine knot structure facilitating receptor (cross-)binding (4).

In physiologic conditions, such as pregnancy, the hCG secreted from the syncytiotrophoblast binds to the LH/CG receptor on the corpus luteum and induces progesterone secretion, thereby maintaining the vascularized endometrium in which the embryo can develop. hCG also stimulates early fetal testosterone secretion in testicular Leydig cells of the male embryo *via* the LH/CG receptor (5).

In conditions of hCG excess, the structural similarities of hCG and TSH (4) may allow hCG to exert an endocrine ‘spillover effect’ on TSH receptors of the thyroid gland, thereby stimulating thyroxine release (6, 7, 8). This phenomenon of cross-reactivity of a hormone with the receptor of another hormone is also referred to as ‘molecular mimicry’. The severity of hyperthyroidism is directly linked to both the amplitude and the duration of excess hCG levels.

In gestational transient thyrotoxicosis (GTT), mild suppression of TSH is observed at the end of the first trimester of pregnancy, with a reverse correlation of β-hCG and TSH but with fT4 concentrations mostly remaining in the normal range (9, 10, 11, 12). This is frequently associated with hyperemesis gravidarum (HG), maternal weight loss or absence of weight gain, tachycardia and unexplained fatigue of pregnant women (13). These symptoms occur without clinical evidence of Graves’ disease. There is evidence that the condition is induced by circulating β-hCG with higher biological activity (10). The clinical course is usually mild and self-limited; only a small proportion of pregnant women experience severe thyrotoxicosis. In twin pregnancies, peak β-hCG concentrations are much higher compared to singleton pregnancies, and they persist much longer, which causes a more frequent and profound decrease in serum TSH, with a transient rise of fT4 serum concentrations (14). However, more recently, fetal production of GDF15 has been demonstrated to contribute to HG, suggesting a more multi-factorial origin of this condition (15).

In gestational trophoblastic disease (GTD), trophoblastic tumours encompassing the premalignant hydatidiform mole and gestational trophoblastic neoplasia (invasive mole, choriocarcinoma and placental site trophoblastic tumour) arise from abnormal proliferation of the placental trophoblast. These tumours secrete large amounts of an hCG variant with increased thyroid-stimulating activity (16). In females with GTD, 7% have biochemical hyperthyroidism, but only 2% manifest with clinical hyperthyroidism. The severity of the disease may vary from a mild course to severe thyroid storm, which potentially leads to multi-organ failure. Removal of the mole or chemotherapy of the carcinoma cures hyperthyroidism (17).

In contrast to these known effects of hCG in pregnant females, reports from males, regarding clinical effects induced by paraneoplastic hCG, are scarce and there have been only a few reports, mostly of male patients with ChCs causing excess β-hCG and thyrotoxicosis.

Herein, we analyse β-hCG and thyroid hormone concentrations in males with hCG-positive testicular GCTs, describe the potential clinical consequences of paraneoplastic hCG secretion and identify high-risk groups for thyrotoxicosis.

## Methods

The study was approved by the Ethics Committee of the University of Münster (approval code: 4 I Nie). Consent has been obtained from each patient or subject after a full explanation of the purpose and nature of the study.

Histopathological testicular sections of *n* = 20 males aged 17–55 years, with GCTs stained positive for hCG, were reviewed to determine their histological composition. In addition, we assessed their β-hCG and thyroid hormone (fT4 and TSH) serum concentrations at presentation, where available. In two individual patients with testicular GCTs who developed symptomatic hyperthyroidism, we describe their clinical course, focussing on differences in therapeutic management and outcome. All data were obtained from retrospective case note review. All patients enrolled in the histopathological study and patient 2 were treated at the University Hospital Münster. Patient 1 was treated at the University Hospital Dresden. At the time of the initial assessment, none of the patients was on thyroid hormone supplementation or anti-thyroid drugs. Due to the retrospective nature of the study, clinical details, such as smoking status, concomitant diseases, weight/BMI and other medication that might affect thyroid function, were unfortunately not available to us.

Gonadal tissues were stained with haematoxylin and eosin (H&E); thereafter, immunohistochemistry (IHC) was performed on 4-μm-thick paraffin sections using the peroxidase-conjugated avidin–biotin method. The primary antibody included a monoclonal mouse β-hCG-antibody. In brief, sections were deparaffinized in xylene and rehydrated through graded ethanol at room temperature. Incubation with the primary antibody was performed for 30 min at room temperature. After washing, the paraffin sections were incubated with biotinylated secondary antibodies.

The following immunoassays were employed to measure hormone serum concentration: TSH: Roche ECLIA, fT4: Roche ECLIA and β-hCG: Roche ECLIA.

## Results

### Histology

**Figure 1:**
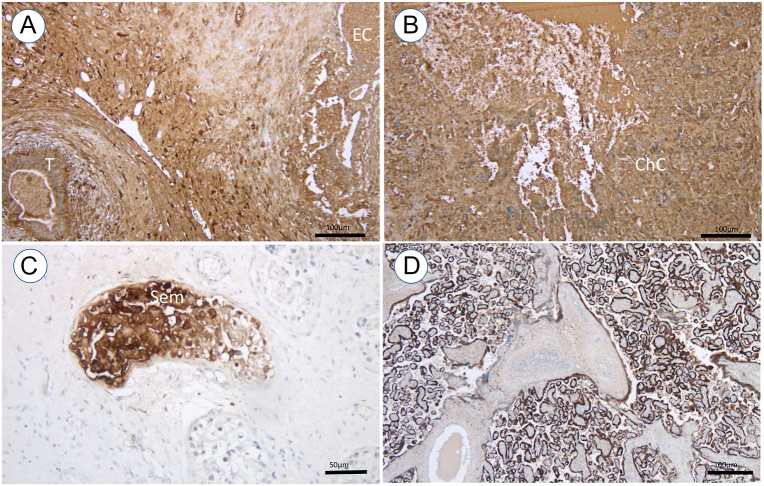
Exemplary immunohistochemistry (IHC) sections of hCG-positive staining, detected by brown precipitation. (A) Mixed testicular tumour consisting of teratoma (T) and embryonic carcinoma (EC). (B) Testicular choriocarcinoma (ChC), (C) testicular seminoma (Sem) and (D) placental tissue used as positive control.

Among *n* = 20 testicular germ cell tumours (GCTs) staining positive for hCG, *n* = 7 were classified as seminomas and *n* = 13 were classified as non-seminomatous (NS) tumours. Of the latter, *n* = 10 had components of an embryonal cell carcinoma (EC) and *n* = 7 had components of a choriocarcinoma (ChC). Figure 1 exemplifies the histopathological images of hCG-producing testicular tumour entities.

### Tumour marker hCG

**Figure 2:**
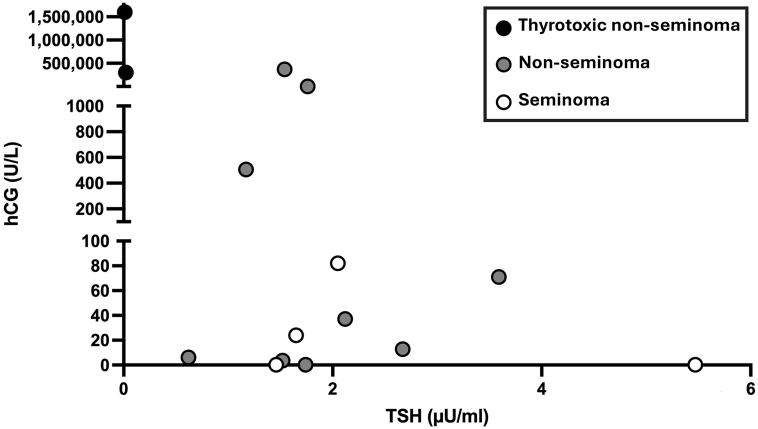
Scatter plot of TSH (*x*-axis) and β-hCG serum levels (*y*-axis) in patients with hCG-producing testicular germ cell tumours from our retrospective analysis of patients with seminomas (open circles), non-seminomatous GCTs (grey circles) and our two patients with non-seminomatous GCTs presenting with thyroid storm (black circles).

hCG serum concentrations were elevated (>0.3 U/L) in only 3/7 males (43%) with seminomas but in 12/13 males (92%) with non-seminomas (Table 1; Fig. 2). In men with hCG-positive seminomas, the mean serum β-hCG concentration was 15.3 ± 30.6, median 0.3 (range: 0.3–82.1) U/L; in men with non-seminomas, the mean serum β-hCG was 228,526 ± 464,596, median 71.1 (range: 0.3–1,600,000) U/L.

**Table 1:** Summary of histological findings, pattern of β-hCG immunostaining and thyroid function tests in our cohort of *n* = 20 men with testicular germ cell tumours.

Age (years)	Tumour entity	β-hCG immunostaining	β-hCG (U/L) at diagnosis	TSH (mU/L) at diagnosis	fT3 (pg/mL) at diagnosis	fT4 (pmol/L) at diagnosis
55	Seminoma	Single cells	82.1	2.05		
21	Seminoma	Partial	24.0	1.65	6.87	13.5
35	Seminoma	Partial	1.9			
51	Seminoma + (syncytio)trophoblast	Partial	0.3			
37	Seminoma	Single cells	0.3	5.47		
40	Seminoma	Single cells	0.3	1.46	5.19	13.4
24	Seminoma	Single cells	0.3		o.d.	o.d.
17 (P1)	Non-seminoma (EC, YS and ChC)	n.a.	1,600,000	<0.01	43.76	>100.0
23 (P2)	Non-seminoma (EC)	Partial	301,396	0.02		
22	Non-seminoma (ChC)	Single cells	370,750	1.54		
27	Non-seminoma (5% EC, 10% YS, 20% S, 25% ChC and 40% T) + seminoma	Partial	693,390			
19	Non-seminoma (40% EC, 35% YS, 20% T and 5% ChC)	Partial	4,635	1.76		
22	Non-seminoma (T, YS and EC) + (syncytio)trophoblast cells	Single cells	506	1.17		
47	Non-seminoma (YS and EC) + seminoma (5%)	Partial	71.1	3.59		
28	Non-seminoma (35% T, 45% EC and 20% YS) + (syncytio)trophoblast	Partial	37.1	2.12		
32	Non-seminoma (10% S, 27% EC, 54% YS and 9% ChC) + seminoma	Partial	27.9	-		
36	Non-seminoma (20% S and 80% T) + seminoma	Single cells	12.8	2.67	5.07	16.47
74	Non-seminoma (EC, ChC and YS) + (syncytio)trophoblast	Single cells	6.2	0.62		
22	Non-seminoma (EC and T)	Partial	3.6	1.52	5.23	18.27
19	Non-seminoma (95% YS and 5% ChC)	Single cells	0.3	1.74	5.42	15.44

ChC, choriocarcinoma; EC, embryonal carcinoma; T, teratoma; YS, yolk sac tumours.

### Thyroid hormones

In two males (10% of all men with hCG-positive GCTs), TSH was suppressed <0.01 mU/L (NR 0.27–4.2) and hCG serum concentrations were excessively elevated (1,600,000 U/L and 301,396 IU/L, respectively). Of two other males with elevated hCG concentrations (370,750 and 693,390 U/L, respectively), one did not have blunted TSH, and in the second, thyroid hormone levels were not available (Table 1; Fig. 2).

### Clinical course of two males with manifest thyrotoxicosis

**Figure 3:**
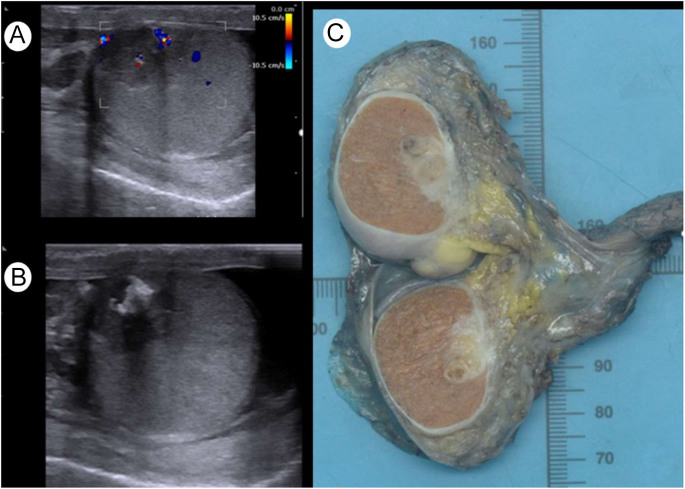
Ultrasound images of the testicular tumour of patient 2 upon presentation (A and B) and macroscopic image of the tumour after surgical resection (C).

The two patients with excessively high β-hCG concentrations (1,600,000 U/L and 301,396 U/L, respectively) were a 17-year-old adolescent (patient 1) and a 25-year-old adult man (patient 2). Both were admitted to the hospital due to the detection of a unilateral testicular mass. An oncologic workup confirmed the diagnosis of an hCG-producing testicular tumour with multiple metastases in retroperitoneal lymph nodes, liver and lung in both patients. Figure 3 illustrates the testicular ultrasound images and the macroscopic tumour in the testis after the orchiectomy in patient 2.

#### Tumour markers in serum

AFP was raised above 7,000 ng/mL in patient 1 and in the normal range in patient 2 (2.9 ng/mL; normal < 7). LDH was elevated in both at diagnosis; in patient 1, it was 35 μmol/L s (normal range: 2.25–3.75), and in patient 2, it was 805 U/I (normal range: 135–225).

#### Clinical symptoms and course

Upon diagnosis of the tumour, both males presented with symptoms of thyrotoxicosis, including agitation and anxiety, tachycardia, abdominal pain, nausea, frequent emesis, weight loss and extreme weakness. In addition, the first patient had fever and severe psychotic symptoms, indicative of thyroid storm; he also presented with significant bilateral gynaecomastia (Tanner B3).

#### Thyroid hormones

**Figure 4:**
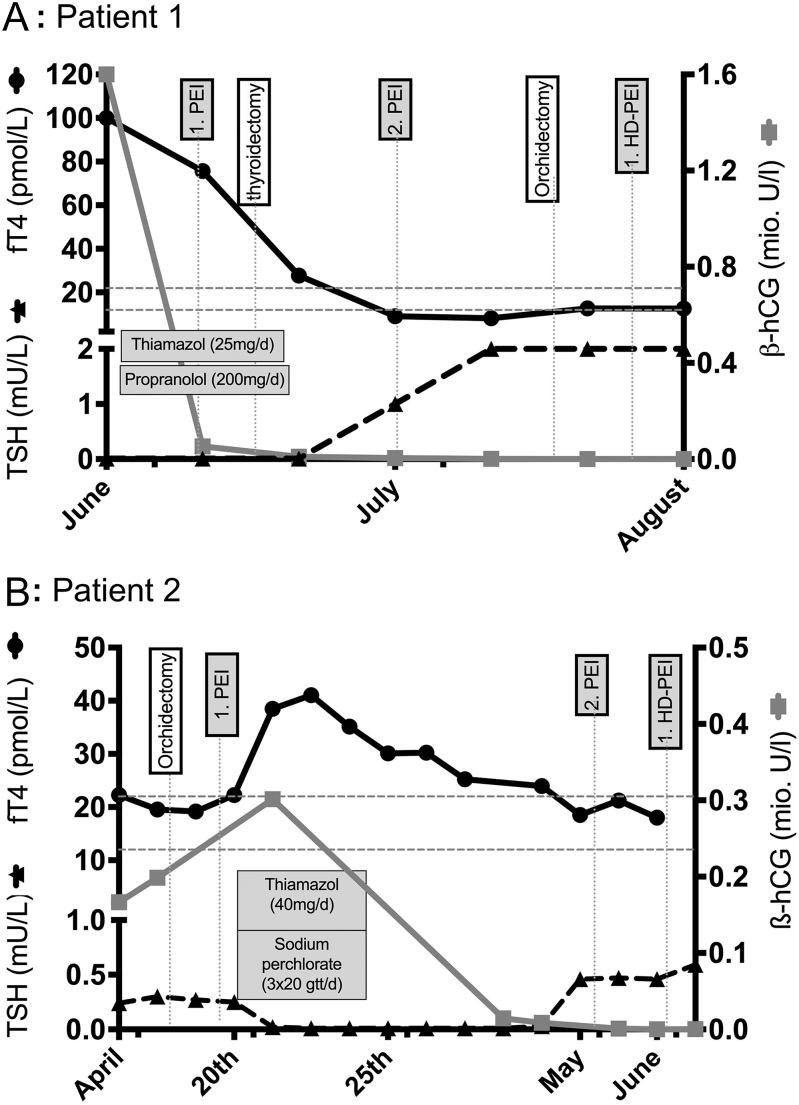
Summary of the clinical course in patient 1 (A) and patient 2 (B) illustrated by their relevant thyroid hormone levels (left *y*-axis) and β-hCG serum levels (right *y*-axis) over the time from presentation until discharge from hospital (*x*-axis). fT4, black circles connected with a black line; TSH, black triangles connected with a black interrupted line; and β-hCG, grey boxes connected with a grey line. The upper and lower fT4 reference range is indicated by the grey horizontal lines. Relevant treatment or interventions are shown in boxes. HD, high dose; PEI, cisplatin, etoposide and ifosfamide chemotherapy.

In both patients, serum TSH concentrations were suppressed below 0.01 mU/L (NR 0.27–4.2) at presentation. While free thyroxine (fT4) levels of >100 pmol/L were measured in patient 1 (Fig. 4A), patient 2 presented with an fT4 concentration of 19 pmol/L, i.e. in the higher normal range (12–22 pmol/L), which increased after one day into the thyrotoxic range (Fig. 4B).

In both patients, the laboratory workup revealed negative autoantibodies for thyroglobulin (TGAb), anti-thyroid peroxidase (TPOAb) and thyroid-stimulating hormone receptor (TSHRAb), excluding Graves’ disease or thyrotoxicosis by autoimmune thyroiditis.

#### Medical treatment

In patient 1, anti-thyroid treatment with thiamazole (25 mg/day) and propranolol (200 mg/day) was started at presentation when thyroid storm was incipient. However, symptoms of hyperthyroidism did not promptly respond to treatment, leading to refractory thyroid storm, although fT4 serum and hCG concentrations started to drop under anti-thyroid medication and chemotherapy (Fig. 4A). Severe associated psychosis and imminent cardiac decompensation led to the decision to perform a total thyroidectomy as a rescue strategy. After that, fT4 serum concentrations fell into the normal range, TSH recovered, and subsequently, thyrotoxic symptoms resolved (Fig. 4A).

In patient 2, hyperthyroxinaemia developed the day after hospital admission. At this time, i.v. thiamazole (40 mg/day) and sodium perchlorate (3 × 20 gtt/day) were administered for 5 days. Nevertheless, fT4 serum concentrations further increased, reaching a maximum of 41.1 pmol/L (NR 12–22 pmol/L) 5 days later, and only normalized 15 days after the initiation of the treatment (Fig. 4B). During this period, fT3 concentrations increased in parallel from 3.5 pg/mL to a maximum of 6.5 pg/mL (normal range: 2–4.4) but remained at hyperthyroid levels only for 4 days before dropping into the normal range. TSH normalized after 20 days of the treatment (Fig. 4B).

#### Surgical treatment, histopathology and tumour staging

Both patients underwent unilateral orchidectomy with histopathological investigation revealing a NSGCT. While the histology of the tumour in patient 1 was classified as a mixed tumour, consisting of parts with EC, yolk sack tumour (YS) and choriocarcinoma (ChC), patient 2 was shown to have a pure EC within the left testis.

Patient 1 was TNM staged ypT1, ypNx, ypMx, L0, V0 and R0 with retroperitoneal lymph node and pulmonal and hepatic metastases. Patient 2 was classified as tumour stage IIIC (according to AJCC 2010), pT1, cN3, CM1b, L0, V0, Pn0 and R0, also with pulmonary and hepatic metastases and retroperitoneal lymph node infiltration below the left renal vein.

#### Chemotherapy

Both patients were initially treated with three-weekly cycles of chemotherapy using PEB/I (cisplatin, etoposide and bleomycin/ifosfamide) and thereafter with three subsequent highly dosed cycles of PEI (according to the MAKAI protocol of the German Society for Paediatric Oncology and Haematology (GPOH) in patient 1). In addition, patient 2 underwent autologous stem cell transplantation.

## Discussion

The present study illustrates that paraneoplastic hCG-production from non-seminomatous testicular germ cell tumours is a rare cause of thyrotoxicosis, which requires early recognition and treatment to avoid life-threatening thyroid storm.

Specifically, our retrospective study demonstrates that ECs and ChCs, which may both be present in NSGCT as a combination, are more likely to produce higher amounts of β-hCG, while seminomas may produce β-hCG only to a reduced extent. Thus, seminomas do not seem prone to affect thyroid function.

The latter observation is in line with a previous study, which included 726 cases with a seminoma. Fifty-two per cent of these patients had elevated β-hCG levels in serum, which was associated with a larger tumour mass (primary tumour and/or metastases). Of note, hCG-positive and hCG-negative seminomas did not have different prognostic outcomes after standard therapy (18).

Our observations contribute to the existing evidence that hCG has thyrotropic activity, which is due to the structural homology of both the hCG and TSH molecules and also their receptors (3, 8). The first clinical evidence for this interaction comes from a case report by Cohen and Utiger (19), who observed that a man with metastatic choriocarcinoma had associated hyperthyroidism. Meanwhile, it is proven that hCG has weak intrinsic activity at the TSH receptor: 1 U hCG ≈ 0.0013 µU hTSH (20). *In vitro*, hCG stimulates iodine uptake, cAMP activity and DNA synthesis in a thyroid carcinoma cell line (21).

Interestingly, we observed that high serum concentrations of paraneoplastic hCG from NSGCTs do not always cause TSH suppression. The phenomenon of different effectiveness of hCG on thyroidal TSH receptors could be explained by individual differences of the β-hCG structure. hCG has a MW of 36.7 kDA (30% carbohydrates) and contains a high proportion of sialic acid. It is possible that abnormal molecular variants of hCG are produced by NSGCTs, containing both hCG molecules with a prolonged half-life and with more potent thyrotropic activity and molecules with a weak action on the TSH receptor. Different variants of hCG contain different amounts of sialic acid. Deglycosylation and/or desialylation of hCG enhances its thyrotropic potency (8). Different thyrotropic activity can also result from changes in the amino acid sequence of the hCG molecule (22).

Our clinical observation is corroborated by a previous study of eight men with non-seminomatous germ cell tumours and with hCG concentrations ranging between 10,000 and 50 million U/L (23), in which no correlation was found between hCG and T4 levels (24). Oosting and coworkers assessed the prevalence of paraneoplastic hyperthyroidism in patients with *metastatic* non-seminomatous germ cell tumours (NSGCTs) (24). Among 144 patients, five with hyperthyroidism (3.5%) were identified, of which all had high serum hCG levels (mean: 1,325,147 IU/L). Half of those had hyperthyroidism, vs 0% of the patients with hCG <50,000 IU/L (*P* < 0.001). The two males in our study with hyperthyroidism also had *metastatic* NSGCT.

By contrast, in another previous study conducted by Goodarzi and Van Herle on hCG and hyperthyroxinaemia in 17 male patients with germ cell tumours aged 16–50 years, a highly significant correlation of serum β-hCG levels between 200,000 and 3.06 million U/L with total T4 was observed (*r* = 0.76; *P* = 0.0068) (25). Giralt and coworkers, who retrospectively evaluated the data of 17 men with GCTs with high levels of β-hCG and with hyperthyroidism, found that in 82% of the cases, the location of the tumour was in the testes, while 18% had *extragonadal* tumours (26). They also observed that serum thyroxine levels correlated with β-hCG levels (*r* = 0.84). All seven patients with elevated T4 levels had β-hCG values greater than 200,000 mIU/mL. Only three had clinical manifestations of hyperthyroidism, and one patient required specific anti-thyroid treatment.

It should be noted that the ‘high-dose hook effect’ can cause false-low measurements of the analyte, in this case, β-hCG (27). The hook occurs if the analyte concentration exceeds the binding capacity of both the capture and the labelled antibodies in the assay reagents. This induces incomplete formation of the immune complexes that are required for adequate detection of the signal. This has to be taken into consideration if a very high concentration of hCG is present, and it is recommended to dilute the sample to avoid false-low measurements. Moreover, tumour lysis during chemotherapy can contribute to β-hCG elevation (28).

Thyroid storm is typically known to occur in Graves’ disease, in patients with toxic nodular goitre, recent use of iodinated contrast or discontinuation of anti-thyroid medications. It is also observed following respiratory tract or gastrointestinal infection (29). Clinical features of thyroid storm include fever, marked tachycardia or tachyarrhythmia, sweating and tremor, nausea and vomiting, diarrhoea, abdominal pain, dehydration (potentially leading to acute pre-renal kidney failure), restlessness, extreme agitation (potentially leading to true psychosis) or coma. Congestive heart failure and respiratory distress are life-threatening complications. Rarely, apathetic storm is observed, with extreme weakness, emotional apathy, confusion and absent or low fever. Death from thyroid storm occurs in 10–25% but can be avoided by prompt recognition and aggressive treatment at an intensive care unit (29, 30, 31, 32). Of note, while free T4 is elevated and TSH levels are suppressed, T3 levels may be markedly reduced in relation to the severity of the illness, as part of the associated ‘non-thyroidal illness syndrome’ (33, 34).

Specific therapy of hyperthyroidism includes the use of increasing doses of beta-blocking agents, such as propranolol, which also inhibits type 1 deiodinase, decreasing the conversion of T4 to T3 or metoprolol. Anti-thyroid drugs include propylthiouracil (PTU) or thiamazole/PTU and may more rapidly reduce circulating T3 levels as they also prevent peripheral conversion of T4 to T3. Sodium perchlorate or potassium iodide is administered one hour after the first dose of thiamazole/PTU.

Primary adrenal insufficiency may develop as the consequence of inadequate cortisol secretion during thyrotoxicosis; it can be treated by adding stress doses of i.v. hydrocortisone. Hydrocortisone also inhibits the conversion of T4 to T3. Rescue strategies include plasmaphaeresis, dialysis and/or thyroidectomy.

Difficulties in the management of patients with metastatic choriocarcinoma complicated by thyrotoxicosis have been a matter for many years (35). In a patient with a mixed germ cell tumour, thyrotoxicosis was even complicated by pulmonary bleeding with ARDS, haemorrhagic brain metastases causing acute stroke, followed by sepsis with renal failure, pancytopenia and death (36).

One difficulty in the management relates to the fact that TSH is used as a screening parameter for thyroid function and is measured as the sole parameter in some hospitals. TSH alone is inadequate to assess thyrotoxicosis, but simultaneous measurement of free thyroid hormone concentrations is required to determine its presence and severity. This is a limitation of our study since a complete set of thyroid function tests was only available in 6/20 patients (Table 1). TSH secretion is regulated according to a set point that reacts with a delay of hours to days to peripheral concentrations of fT4 and T3, and this may impair prompt diagnosis of incipient hyperthyroidism (37, 38). In addition, TSH levels can be low despite normal concentrations of free thyroid hormone levels, as seen in subclinical hyperthyroidism, often due to intermittent systemic sickness disease or drug-induced alterations of the hypothalamic–pituitary–thyroid axis (39). Although we ruled out concomitant thyroid supplementation or anti-thyroid medication in our cohort, further clinical details were not available to us due to the retrospective nature of our study.

## Conclusions

Thyrotoxicosis is a rare complication of paraneoplastic hCG production in males with non-seminomatous germ cell tumours. A low threshold of suspicion should be maintained for the possibility of hyperthyroidism in patients at high risk of paraneoplastic hyperthyroidism and thyrotoxicosis, i.e. patients with testicular choriocarcinoma or embryonal cell carcinoma with elevated serum concentrations of hCG. Early recognition and early anti-thyroid treatment of incipient hyperthyroidism, combined with orchiectomy and concurrent chemotherapy, can decrease hCG levels and concomitantly hyperthyroxinaemia and may prevent life-threatening thyroid storm.

The development of hyperthyroidism is largely influenced by the level of β-hCG and the molecular structure of the hCG. However, which isoform exhibits a higher thyrotropic potency is still unresolved. Its identification could have an impact on the prediction of hyperthyroidism associated with β-hCG-producing tumours.

## Declaration of interest

The authors declare that there is no conflict of interest that could be perceived as prejudicing the impartiality of the work.

## Funding

This work did not receive any specific grant from any funding agency in the public, commercial, or not-for-profit sector. AH is supported by the Deutsche Forschungsgemeinschaft (DFG, 314061271-TRR/CRC 205).
